# A modern overview of the process of platelet formation (thrombocytopoiesis) and its dependence on several factors

**DOI:** 10.11613/BM.2024.030503

**Published:** 2024-10-15

**Authors:** Anastasia Ivanovna Gabrilchak, Oksana Anatolievna Gusyakova, Vladimir Aleksandrovich Antipov, Elizabeth Alekseevna Medvedeva, Lyubov Leonidovna Tukshumskaya

**Affiliations:** 1 Professional Center for Education and Research in Genetic and Laboratory Technologies, Samara State Medical University of the Ministry of Health of the Russian Federation, Samara, Russia; 2 Department of Fundamental and Clinical Biochemistry with Laboratory Diagnostics, Samara State Medical University of the Ministry of Health of the Russian Federation, Samara, Russia; 3 Institute of General Medicine, Samara State Medical University of the Ministry of Health of the Russian Federation, Samara, Russia; 4 Institute of Pediatrics, Samara State Medical University of the Ministry of Health of the Russian Federation, Samara, Russia

**Keywords:** megakaryocyte, platelet, microenvironment, cytokines, apoptosis

## Abstract

Structural and functional alterations in platelets are an actual problem that requires more attention. The treatment of these illnesses proves challenging, inefficient and heavily relies on platelet donations. A difficult task confronting science is producing platelets *in vitro*, which calls for meticulous examination of factors affecting platelet generation. It is known that megakaryocytes produce platelets *in vitro* and *in vivo* differently: in the laboratory we can get a smaller number of platelets compared to the human body. This review primarily examines the stages of megakaryocyte maturation and the processes involved in platelet formation. The article reflects the results of both fundamental research on the problem and the new results obtained over the past decade. Currently, most scientists accept the pro-platelets theory of platelet formation. This review aims to explore in detail each stage of pro-platelet formation and the platelet formation process. It explains on the processes of polyploidization, endomitosis, and apoptosis, as well as the functions of structural cell components (microtubules, mitochondria, T- and α-granules) and pro-platelet migration. The microenvironment influence is acknowledged for the osteoblastic and vascular niches that affect thrombocytopoiesis. The additional aspect is the contribution of specific proteins to thrombocytopoiesis such as RhoA, β1-tubulin, cytokines IL-6, IL-8, Toll-like receptors, *etc*.

## Graphical abstract

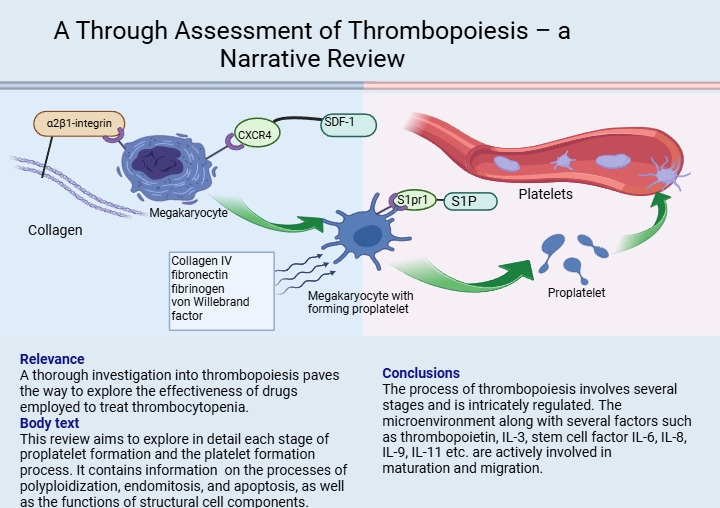

## Highlights

• Thrombocytopoiesis is a multistep process

• The microenvironment is actively involved in the maturation and migration of megakaryocytes

• During maturation, megakaryocytes undergo a process of polyploidization

## Introduction

Throughout history, platelets have piqued the interest of researchers from their discovery until the present day. Platelets that were not consumed in the formation of blood clots or other (patho)physiological processes, circulate in the bloodstream for 7-10 days, and are then destroyed in the liver. Approximately 1/3 of platelets are permanently stored in the spleen (*1*). In individuals aged six years and above, normal platelet counts are independent of sex and range from 150-400 x10^9^/L cells (*1*). Whereas, children from birth to six years of age are distinguished by their own reference values, which are dependent upon both sex and specific age. There are several types of platelet-associated pathological conditions, among which are: thrombocythemia (an increase in the number of platelets), thrombocytopenia (a decrease in the number of platelets) and platelet dysfunction (*1*). Thrombocytopenia is one of the most acute problems. Infections, malignant neoplasms, liver diseases, autoimmune diseases and blood clotting disorders are identified as the main causes of thrombocytopenia. The patient observes symptoms such as constant fatigue, headache, bleeding, breathlessness, unexplained bruising, which occur with a drop in platelet count to below 100 x10^9^/L (*2*). The prevalence ranges from 10-130 to 200 *per* 1 million people (*3*). Platelet transfusion is a therapeutic option for the treatment of this pathology, in addition to drug treatment, such as hormone therapy (*e.g.* corticosteroids), taking thrombopoietin receptor agonists (Romiplostim, Eltrombopag) and splenectomy (*4*). One of the most commonly used drugs for the treatment of thrombocytopenia is Romiplostim. It binds to and activates thrombopoietin receptors on megakaryocyte precursors in the bone marrow. Drug binds in the same way as endogenous thrombopoietin (TPO) and can displace TPO from its receptor. Romiplostim activates numerous pathways analogous to those stimulated by TPO, resulting in sustained increasing platelet counts in patients with thrombocytopenia (*5*).

However, the life of isolated platelet is only 24 hours, in a thrombomixer up to 5 days, which is an extremely short period. The dependence of patients on donated blood is another problem on the way to overcoming the disease, while the number of donors is decreasing annually (*6*). Today, researchers face a new task - the creation of platelets *in vitro* for the constant production of the required amount of platelet mass.

A thorough investigation into thrombocytopoiesis paves the way to explore the effectiveness of drugs employed to treat thrombocytopenia. However, while scientists are searching for ways to put this idea into practice, it is necessary to scrutinize every stage of platelet formation and the mechanisms that determine not only the physiological state, but also pathology, as well as the conditions that affect the speed of these processes.

Thus, the purpose of our review is to summarize current data on the process of platelet formation and the influence of various factors on this process. Currently, there are two theories that describe the formation of platelets - fragmentation (theory of demarcation membranes) and the theory of pro-platelets. The fragmentation theory describes that the membrane increases its area by invagination and divides the cytoplasm into “fields”. The megakaryocyte, in the process of thrombocytopoiesis during migration from the bone marrow to the lungs, laces platelets whose outer membrane is the demarcation membrane of the megakaryocyte. However, reliable evidence of this theory has not been presented and requires further study (*7*). The second theory was confirmed after the recording of the pro-platelets formation process by Italiano *et al.* which is an irrefutable proof of its existence (*8*). In this review, we will consider only the theory of pro-platelets. However, it should be noted that each of the described theories could potentially be realized in the context of the organism (*8*).

Three stages can be distinguished in platelet formation: differentiation of a hematopoietic stem cell into a megakaryocyte, its endomitosis with polyploid formation, and platelet release. Each stage has its own characteristics and plays a huge role in the formation of mature platelets (*9*).

## Differentiation of a hematopoietic stem cell into a megakaryocyte

Megakaryocytes develop in the bone marrow from precursor cells - hematopoietic stem cells; their number is less than 0.05% of the total number of nucleated cells in the bone marrow. They can also be found in the yolk sac, spleen and liver of the fetus in the early stages of intrauterine development (*10*). They asymmetrically differentiate into multipotent precursor cells, gradually lose the ability to self-renew and pluripotency for the regeneration of megakaryocytic or erythroid precursors. Thrombopoietin takes an active part in this process by transmitting signals through the thrombopoietin receptor c-MPL, which is expressed at all stages of thrombocytopoiesis from the hematopoietic stem cell to the platelet. GPIba (plasma membrane glycoprotein complex or von Willebrand factor platelet receptor) also regulates the synthesis of thrombopoietin in the liver as part of the acute phase reaction, which is controlled by interleukin 6 (IL-6), and helps stimulate thrombocytosis in systemic inflammation. In mice, IL-1a can cause a new mechanism of “megakaryocyte rupture” (cytoplasm separation), which promotes emergency thrombocytopoiesis (*11*).

The complex of thrombopoietin with cMPL activates Janus kinase 2 (JAK2), which attracts signal transducers and transcription activators (STATs), phosphoinositide-3-kinase (PI3K) and mitogen-activated protein kinase (MAPK) (*12*).

The final stages of megakaryocytopoiesis remain inadequately understood. Nevertheless, research confirms that interleukins and stem cell factors have a direct effect on platelet production *in vitro*, and also induce the formation of pro-platelets (*13*). Cytokines such as IL-3, stem cell factor (also known as kit ligand or SCF), IL-6, IL-8, IL-9, IL-11, IL-1-alpha, Flt-3 ligand (FL or fms-like tyrosine kinase 3) are also involved in the process of megakaryocytopoiesis and thrombocytopoiesis. In addition to the cytokines listed above, the process involves tyrosyl-tRNA synthetase (YRSACT), chemokine ligand 5 C-C motif (CCL5), also known as RANTES (cytokine synthesized by normal T-lymphocytes), insulin-like growth factor-1 (IGF-1), stromal cell factor-1 (SDF-1) and fibroblast growth factor 4 (FGF-4) (*14*).

Resting platelets express glycoprotein 130 (gp 130) on their membranes, and trans signaling occurs in the presence of IL-6, which increases platelet production (*15*). On the surface of the platelets themselves, there are many receptors that are associated with the G-protein, CXCR1 and CXCR2 are able to interact with IL-8. Interleukin 8 stimulates procoagulant activity, causing platelet production (*16*). Interleukin 6 with low doses of IL-3 increases the number of immature megakaryocytes and stimulates their development. Consequently, IL-6 and IL-8 affect clotting profiles and trigger platelet hyperactivation.

As is known, erythropoietin, in addition to stimulating erythropoiesis, also activates acetylcholinesterase and increases DNA synthesis, which leads to the activation of cytoplasmic processes and the production of pro-platelets (*17*).

Recently, researchers have identified hematopoietic stem cells (HSCs) with a bias towards the megakaryocyte line, which directly generate unipotent precursors limited by megakaryocyte to rapidly replenish mature cells. The limited megakaryocyte precursor has a critical role in response to the acute need for platelets (*18*).

## Endomitosis and polyploidization of megakaryocytes

During the maturation of the megakaryocytes in preparation for thrombocytopoiesis, there is an increase in cytoplasmic cytoskeletal proteins and platelet-specific granules, and an invaginated membrane system develops. The maturation of megakaryocyte regulates thrombopoietin, with its help, megakaryocyte becomes polyploid (*19*).

During the process of differentiation, megakaryocytes undergo an incomplete cell cycle repeatedly. This cycle is characterized by the interruption of mitosis in late anaphase, affecting both karyokinesis and cytokinesis, and is called endomitosis. Megakaryocytes first undergo a proliferative 2N stage, in which their proliferation through the cell cycle is identical to other hematopoietic cells. Megakaryocytes then undergo endomitosis and accumulate content of 4, 8, 16, 32, 64 and 128N DNA in one polyploid nucleus before continuing their final maturation and subsequent formation of pro-platelets (*20*).

The effectiveness of endomitosis is achieved through various mechanisms. The primary mechanism involves the inhibition of Aurora B kinase, a significant enzyme in the process of chromosome segregation. It is a serine/threonine kinase that localizes to the telomeres of stem cells and interacts with the essential telomeric protein CRF1 of the kinetochore. It attaches the mitotic spindle of division to the centromere (*21*). Megakaryocytes have been shown to express the functional kinase Aurora B during endomitosis (*22*). This inhibition is partially caused by phosphorylation by the Gase protein, which activates the serogroup of the Rho protein, MgcRacGAP, which, in turn, changes the activity of a protein member of the Ras A homologue family (RhoA) (*23*).

The transition from mitosis to endomitosis corresponds to the late stage of cytokinesis. During endomitosis, the formation of the central sulcus occurs typically, but the contractile ring differs as there is no accumulation of non-muscular myosin IIA (*24*). In order to initiate the transition from mitosis to endomitosis, transcription factor 1 (RUNX1) suppresses the heavy chain of myosin IIB (MYH10). The suppression of the guanine nucleotide exchange factor H1 (GEF-H1) is a mechanism that inhibits the fission furrow, which is responsible for the activation of RhoA. RhoA regulates the actin cytoskeleton, and as a result, cytokinesis is not completed. Next, the transforming sequence of epithelial cells (ECT) participates in polyploidization, which regulates the activation and localization of RhoA (*25*).

Generally, a pure cytokinesis defect results in a multinucleated cell, except for megakaryocytes characterized by a single polylobular nucleus. The analysis of nuclear kinetics in endomitosis revealed the presence of nucleoplasmic bridges in most multipolar cells of endomitosis from telophase to cytokinesis failure. This fact indicates that megakaryocyte endomitosis is a karyokinesis defect, which explains why polyploid megakaryocytes exhibit a single polylobular nucleus along with an increase in ploidy (*26*).

RhoA GTPase has a varied impact on the maturation of megakaryocytes. The RhoA pathway plays a central role in the formation of the contractile ring during cytokinesis. Active RhoA regulates actin polymerization and myosin activation in the median zone through interaction with various effectors (*27*).

Inhibition of Rho kinase (ROCK) transmission leads to an increase in polyploidization in megakaryocytes obtained from umbilical cord blood (*28*). The elimination of RhoA in megakaryocytes *in vivo* led to a significant macrothrombocytopenia. Such megakaryocytes were larger, had higher ploidy and denser membranes with microtubule invagination (*29*). Consequently, the guanine exchange factor H1 (GEF-H1) and epithelial cell transforming factor 2 (ECT 2), which are critical for the activation of RhoA during cytokinesis, should be reduced for polyploidization of megakaryocytes (*23*).

There is a hypothesis that megakaryocytes ploidy is necessary for the synthesis of a considerable amount of mRNA and protein, which are required for platelet packing and performing multiple other functions (*30*). However, the correlation between the amount of DNA and the quantitative formation of thrombocytopoiesis is still being discussed. For example, nicotinamide, which is used to increase ploidy in megakaryocytes during mouse experiments, has shown controversial results. One group observed an induced increase in pro-platelets, while the other group observed a decrease (*17*, *31*). However, platelet counts remained unchanged in both cases (*32*).

It is assumed that despite the inhibition of the fission furrow, there is a process that regulates repetitive DNA replication cycles. Cyclin-dependent kinases are protein kinases characterized by the need for a separate subunit – cyclin. It provides the domains that are necessary for enzymatic activity. These kinases play an important role in the control of cell division and transcription in response to several extracellular and intracellular signals (*33*). Since varieties of cyclin proteins are involved in the regulation of megakaryocyte endomitosis, such as cyclin D-type, cyclin E and cyclin A, some cyclin-dependent kinase inhibitors also act as fixators in endomitosis and include p19INK4D (CDK4 inhibitors) and p21Cip1 (cyclin-dependent kinase 1 inhibitor)/Waf1 (*34*-*38*). This process confirms the hypothesis that the components of the G1 phase are important for stimulating an increase in the ploidy of the megakaryocyte (*35*).

Endomitosis also plays an important role in the creation of an invaginated membrane system (IMS). The IMS is a complex of cisterns and tubules that is distributed throughout the cytoplasm of a megakaryocyte and is a continuation of the plasma membrane as a membrane reservoir for the formation of pro-platelets. A group of researchers confirmed IMS as a source of pro-platelets and platelets. Moreover, the ability for IMS to perform internal migration is reliant on the formation of actin filaments through the Wiskott-Aldrich syndrome (WASP-WAVE) protein pathway on the cytoplasmic side of IMS in response to phosphatidylinositol 4,5-bisphosphate signaling (*36*).

## Granule formation and platelet release

After the endomitosis process, already mature polyploid megakaryocytes form long branching pro-platelets that enter into sinusoidal blood vessels of the bone marrow with the help of β1-tubulin. The pro-platelets are outgrowths of cells that consist of vesicles linked by cytoplasmic bridges. Thus, the cytoskeleton is one of the important components of the formation and growth of pro-platelets. In mice lacking β1-tubulin, structural defects of microtubules and a decrease in the number of spiral microtubules on the periphery of platelets are detected, which leads to a reduction in platelet production by 60% (*39*).

In addition to pro-platelets and platelets, pre-platelets were identified within the megakaryocyte culture. These are discoid cells that lack a nucleus and measure between 2 and 10 μm in diameter. They can be transformed reversibly into barbell-shaped pro-platelets. Conversion from pre- to pro-platelets is controlled by microtubule-based forces, which are determined by two fundamental biophysical properties: microtubule helix diameter and thickness (*40*).

Researches have shown that mitochondrial fusion or division dynamics play a critical role in energy production, cell division, differentiation, and apoptosis. Recent research has indicated that platelet formation and the final maturation of megakaryocytes are triggered by mitochondrial reactive oxygen species (ROS). In addition, it was found that megakaryocyte distortion is associated with ROS levels in mitochondria and, consequently, with platelet formation. This finding agrees with prior studies where ROS impacted the reorganization of the cytoskeleton (*41*).

Microtubules, in addition to their main function, are also involved in the transportation of organelles and granules into platelets and platelet assembly. Vesicles carrying granular cargo are split off from the Golgi network and transported to multivesicular bodies, where proteins are sorted and packed into granules which are then dispatched from the megakaryocyte body to pro-platelets. F-actin forms assembly points in all pro-platelets, resulting in multiple bends and bifurcations (*42*). Cytoplasmic elements move from the megakaryocyte body to pro-platelets in both directions until they are captured by it. This process is based on the bipolar arrangement of microtubules inside pro-platelets (*14*).

During the process of megakaryocyte differentiation, specific platelet-specific granules, responsible for their various functions, are formed and accumulated. This occurs simultaneously with megakaryocyte endomitosis. Mature platelets contain abundant reserves of secretory vesicles, which include dense granules, lysosomes, T-granules and α-granules (the latter is 50-80 *per* platelet) which transfer endogenously synthesized or endocytotic material. Vesicles with granular cargo are detached from the Golgi network and transferred to multivesicular bodies, where proteins are sorted and packed into granules, which are then dispatched from the megakaryocyte body to pro-platelets due to microtubules. Dense δ-granules contain membrane transporters, high concentrations of calcium, bioactive amines, adenine nucleotides and polyphosphate. Lysosomes contain enzymes which are involved in the breakdown of proteins, carbohydrates, and lipids. T-granules are so-called because their discovery was based on the localization of toll-like receptor 9 (TLR-9) (*43*). Several GTP-binding proteins have been shown to play critical roles in the biogenetics of megakaryocyte dense granules, including Rab38, Rab27a and Rab27b (*44*, *45*).

α-granules are formed during megakaryocyte maturation and are the most abundant granules in platelets. Immature megakaryocytes appear to contain only a few mature α-granules and a large number of α-granular precursors (*46*, *47*). Upon activation, platelets release a multitude of factors that help mediate their dynamic functions in hemostasis, inflammation, tumor metastasis and angiogenesis. The majority of these bioactive molecules are released from α-granules, which are unique to platelets and contain an incredibly diverse set of cargoes, including integral membrane proteins, procoagulant molecules, chemokines, mitogenic, growth and angiogenic factors, adhesion proteins and antimicrobial proteins (*48*).

Platelets with α-granule deficiency characterize “grey platelet syndrome”, a rare inherited disorder, making it a good model to study α-granule biogenesis in megakaryocytes. In this pathology, thrombocytopenia is moderate, and the absence of α-granule content gives platelets a typical grey appearance during blood sample microscopy (*49*). VPS33B and Sec1/Munc18 proteins have been shown to be involved in the formation of intracellular vesicles, which is important for α-granule maturation in megakaryocytes but not for their secretion (*50*). The VPS16B protein has been identified as a VPS33B-binding protein and is also required for platelet α-granule development (*51*). Dense δ-granules belong to a family of tissue-specific, lysosome-associated organelles, that originate from the early endosomes of megakaryocytes (*52*).

Several mechanisms play an important role in the proliferation of pro-platelets in the vascular space, including pressure induced by blood flow movement and the operation of podosomes. Podosomes, located on the outer surface of the megakaryocyte plasma membrane, actively degrade the extracellular matrix, cleaving fibrinogen and thereby extending the pro-platelet bulge across the basal membrane. The megakaryocyte forms a podosome by actin polymerization through the Arp2/3 complex (actin-related proteins) and WASP (Wiskott-Aldrich syndrome protein) (*53*).

The movement of blood created by pressure helps to separate pro-platelet fragments from the body. This means that fluid shear forces in the sinusoids of the bone marrow facilitate the intravascular release of fragments protruding from mature megakaryocytes (*54*).

Pre-platelets are released into the bloodstream and undergo rapid and spontaneous transformation into rod-shaped pro-platelets, with a morphology resembling a barbell. This transformation leads to the development of mature platelets. Alternatively, pro-platelets may enter the microcapillaries of the bone marrow, lungs, or spleen, where the shear forces of blood flow cause pro-platelets to produce platelets. Terminal platelet formation occurs within the lungs. In peripheral blood, pre-platelets are typically present in very small numbers or absent, as they undergo further maturation and transformation into mature platelets that circulate in the bloodstream (*55*).

Studies on humans have consistently shown that platelet concentrations are higher in the pulmonary vein than in the pulmonary artery, indicating intrapulmonary thrombocytogenesis. To prove this theory, a series of experiments involving mice were developed. To do this, the process was visualized by *in vivo* two-photon microscopy, providing a rough estimate that half of mouse platelets are generated in the lungs (*56*).

While intravascular and interstitial megakaryocytes were both identified, platelet production was mainly mediated by megakaryocytes of extrapulmonary origin that were deposited in the pulmonary capillary bed. It should be noted that the proportion of platelets produced in the lungs is currently a topic of debate among the scientific community, with the exact proportion of human platelets produced in the lungs remaining unestablished. Some reports suggest that platelet biogenesis in the lungs is about 10 million cells *per* hour (*57*). However, as described above, ROS has a direct effect on pro-platelet production (*41*).

Larger platelets possess a greater pro-platelet potential. Increased platelet size is associated with increased platelet aggregation and adhesion molecule expression, as well as an elevated risk of cardiovascular and peripheral arterial disease. The process of thrombocytopoiesis in the bone marrow is efficient enough to satisfy incessant platelet demand: each megakaryocyte releases approximately 10^4^ platelets (*58*).

## Role of apoptosis in thrombocytopoiesis

Apoptosis also plays a vital role in thrombocytopoiesis. Initially, apoptosis was perceived as exclusively programmed cell death in the process of natural aging. megakaryocytes were assumed to undergo apoptosis and be eliminated by macrophages immediately after releasing functional platelets (*59*). Nevertheless, it has now been revealed that the role of apoptosis in the megakaryocyte is more complicated. Studies were conducted on cord blood, peripheral blood and bone marrow cells and revealed that apoptosis occurs only in mature megakaryocytes and coincides with platelet detachment from the megakaryocyte membrane. The formation of functional platelets also coincides with physiological changes in the cell alike to apoptosis, such as condensation of plasma and nuclear membranes, disruption of cytoskeletal architecture, cell wrinkling and packing, and DNA fragmentation (*14*).

The initiation of apoptosis and platelet formation, depends on the activation of a number of cysteine-aspartate proteases known as caspases. It is also believed that platelet production is independent of the intrinsic and extrinsic megakaryocyte apoptosis pathways, and the intrinsic apoptosis pathway must be inhibited during platelet formation (*60*).

Two general pathways of apoptosis have been described: one intrinsic and one extrinsic. The intrinsic, or ‘mitochondrial’ pathway is controlled by a number of proteins, including B-cell lymphoma 2 (Bcl-2), B-cell lymphoma-extra-large (Bcl-xL), B-cell lymphoma-w (Bcl-w) and Myeloid cell leukemia-1 (Mcl-1), which act to regulate the process, and Bcl-2 antagonist killer 1 (Bak), Bcl-2-associated X protein (Bax) and Bcl-2 related ovarian killer (Bok), which act to induce apoptosis. If improperly controlled, Bak and Bax form oligomers and penetrate the outer mitochondrial membrane. This results in the release of cytochrome c, which activates apoptotic protease-activating factor 1 (Apaf-1). Upon activation, Apaf-1 attracts procaspase-9 to form the apoptosome. This initiates a cascade of events that ends up in the activation of caspases-3 and -7. These caspases are ultimately responsible for the apoptosis of the cell by cleaving various substrates. The extrinsic pathway involves the binding of ligands to TNF receptors. This leads to activation of caspase-8, which activates caspase-3 independently of the intrinsic pathway (*61*).

A series of experiments involving mice with deletion of a gene associated with the intrinsic apoptosis pathway were designed, and they were used to study the relationship between the intrinsic apoptosis pathway and platelet production. Mice with the Bcl-2-deficient megakaryocyte line exhibited normal platelet production and platelet lifespan. Deletion of Bcl-2 and Mcl-1 (induced myeloid leukemia cell differentiation protein) in megakaryocytes had no significant effect on platelet production or survival. These data suggest that Bcl-2 and Mcl-1 may not be required for megakaryocyte and platelet development and survival (*15*).

Thus, there is considerable evidence that apoptosis has a positive effect on thrombopoiesis, which activates megakaryocyte to stimulate plasma protein fractions and platelet release. Nonetheless, the exact mechanism of this pathway remains unclear.

## The microenvironment and its role in thrombocytopoiesis

**Figure 1:**
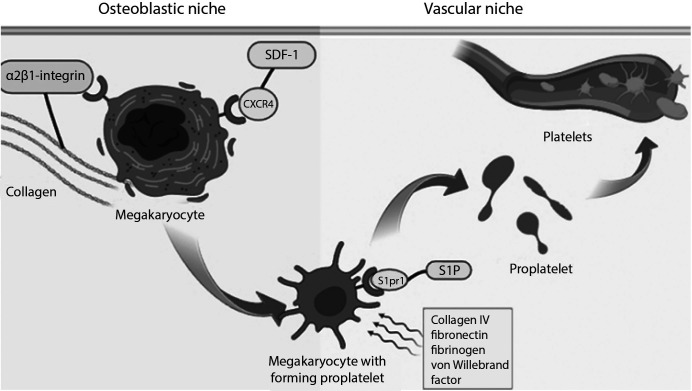
Effect of microenvironment on platelet release.

The microenvironment is a specific complex of cellular structures including reticular, adipose, osteogenic, adventitial cells, macrophages, and components of the extracellular matrix, such as collagen and fibronectin. *In vitro* research reveals that megakaryocytes in an environment consisting only of the solutions for experiments produce pro-platelets at a much lower rate than those in close proximity to bone marrow *in vivo*. This suggests that the megakaryocyte microenvironment has a significant influence on pro-platelet and subsequent platelet production rates (*62*). Megakaryocytes interact with extracellular matrix proteins in the bone marrow that promote megakaryocyte development and subsequent release into the vascular niche. This is because pro-platelet formation cannot occur in the osteoblast niche. The megakaryocyte binds to collagen I, which is the most abundant component of the osteoblast niche, *via* α2β1-integrin. This interaction inhibits pro-platelet formation and leads to full maturation and growth of the megakaryocyte. In mature polyploid megakaryocytes, increased expression of the CXCR4 receptor occurs under the influence of vascular endothelial growth factor (VEGF) (*63*). Furthermore, megakaryocytes influenced by cytokine SDF-1 (SDF-1 is synthesized by bone marrow stroma cells for localizing hematopoietic progenitor cells in their niche and preventing premature release from bone marrow) bind through CXCR4, before leaving the osteoblast niche to localize in sinusoidal endothelial cells. This initiates the formation of pro-platelets which then release platelets into the intravascular sinusoidal space. The vascular niche, composed of collagen IV, fibronectin, fibrinogen and von Willebrand factor, promotes the process of thrombocytopoiesis (Figure 1).

The morphology pro-platelet ensures the transportation of platelets into the bloodstream. S1P (sphingosine-1 phosphate) and megakaryocyte’s receptor (S1pr1) play key roles in pro-platelet elongation and release. Upon contact with blood, S1P acts on the pro-platelet, resulting in platelet release. Platelets produce S1P, which they store and release into the bloodstream when activated by ADP and thrombin. Researches have shown that the unique role of S1P and its receptor S1pr1 play a distinctive role as the main regulators of thrombocytopoiesis. S1P controls two key steps in the megakaryocyte thrombocytopoiesis cascade, including the polarized development of pro-platelet expansions into the bloodstream and their subsequent release from transendothelial stalks. Therefore, the absence of S1pr1 is incompatible with physiological thrombocytopoiesis (*64*).

## Conclusion

The process of thrombocytopoiesis, starting from hematopoietic stem cell maturation to platelet release into the bloodstream, involves several stages and is intricately regulated. The microenvironment (osteoblast niche) along with several factors such as thrombopoietin, IL-3, stem cell factor, IL-6, IL-9, IL-11, *etc.* are actively involved in megakaryocyte’s maturation and migration. During maturation, the megakaryocyte undergoes a process of polyploidization and increases its DNA exponentially. As a result, polyploid megakaryocytes under the action of β1-tubulin form long outgrowths (pro-platelets). These are under the influence of external and internal factors, including reactive oxygen species, F-actin, accumulation of T- and α-granules, apoptosis, blood flow movement pressure and microenvironment, release platelets into the bloodstream.

## Data Availability

All data generated in the presented study are included in this published article.
